# Love in the time of Zoom: how intimacy modulates brain and behaviour synchrony in face-to-face versus video communication

**DOI:** 10.1093/scan/nsaf070

**Published:** 2025-07-10

**Authors:** Xia Wu, Yue Hao, Shuoxian Zhang, Huan Zhang, Yunpeng Jiang, Ying Chen, Zong Zhang

**Affiliations:** Key Research Base of Humanities and Social Sciences of the Ministry of Education, Academy of Psychology and Behavior, Tianjin Normal University, Tianjin, 300387, China; Faculty of Psychology, Tianjin Normal University, Tianjin, 300387, China; Tianjin Key Laboratory of Student Mental Health and Intelligence Assessment, Tianjin, 300387, China; Key Research Base of Humanities and Social Sciences of the Ministry of Education, Academy of Psychology and Behavior, Tianjin Normal University, Tianjin, 300387, China; Key Research Base of Humanities and Social Sciences of the Ministry of Education, Academy of Psychology and Behavior, Tianjin Normal University, Tianjin, 300387, China; Key Research Base of Humanities and Social Sciences of the Ministry of Education, Academy of Psychology and Behavior, Tianjin Normal University, Tianjin, 300387, China; Faculty of Psychology, Tianjin Normal University, Tianjin, 300387, China; Tianjin Key Laboratory of Student Mental Health and Intelligence Assessment, Tianjin, 300387, China; Key Research Base of Humanities and Social Sciences of the Ministry of Education, Academy of Psychology and Behavior, Tianjin Normal University, Tianjin, 300387, China; Faculty of Psychology, Tianjin Normal University, Tianjin, 300387, China; Tianjin Key Laboratory of Student Mental Health and Intelligence Assessment, Tianjin, 300387, China; School of Vocational Education, Tianjin University of Technology and Education, Tianjin, 300387, China; Key Research Base of Humanities and Social Sciences of the Ministry of Education, Academy of Psychology and Behavior, Tianjin Normal University, Tianjin, 300387, China; Faculty of Psychology, Tianjin Normal University, Tianjin, 300387, China; Tianjin Key Laboratory of Student Mental Health and Intelligence Assessment, Tianjin, 300387, China

**Keywords:** interpersonal neural synchrony, hyperscanning, fNIRS, video-mediated communication, intimacy, behavioral coordination

## Abstract

This study explores how video-mediated communication (VMC) and face-to-face communication (FTF) affect social bonding in relationships of varying intimacy. Using hyperscanning fNIRS and dyadic behavioural analysis, data from 72 dyads (36 romantic couples and 36 friends) were analysed. Results revealed an intimacy-by-modality interaction: couples showed better behavioural coordination and higher interpersonal neural synchrony (INS) in the 0.081–0.09 Hz band during FTF, while friends had better synchrony in VMC. In the 0.038–0.046 Hz band, friends exhibited greater INS during FTF, and couples showed better synchrony in VMC. These patterns suggest that high-frequency INS is associated with real-time social cue integration, while low-frequency INS relates to ongoing relational monitoring. Verbal behaviour fully mediated the relationship between satisfaction and FTF-induced prefrontal INS, highlighting connections among psychological states, behaviour, and neural alignment. Granger causality analysis showed a female-to-male neural influence during FTF, absent in VMC, likely due to reduced nonverbal signals. These results demonstrate that the influence of video mediation on interpersonal synchrony is relationship-specific and frequency dependent, empirically supporting a relational-context model that links attachment-based sensorimotor tuning with channel-selection processes in the Communicative Interdependence Perspective.

## Introduction

Human communication is a cornerstone of social bonding, facilitating the exchange of emotions, intentions, and shared understanding through the dynamic integration of verbal and nonverbal cues (Frith and Frith 2006, Hari et al. 2015). Neurobiologically, interpersonal interaction engages distributed cortical networks governing social cognition. Functional neuroimaging studies reveal that face-to-face conversation (FTF) recruit the prefrontal cortex (PFC) for executive control and goal alignment, the temporoparietal junction (TPJ) for mentalizing and perspective-taking, and the mirror neuron system for embodied synchronization of gestures and emotions (Koike et al. 2015, Redcay and Schilbach 2019). The synergistic operation of these neural systems facilitates the social-cognitive functions of neurocomputational processes fundamental to relational bond maintenance and dyadic homeostasis (Hasson et al. 2012). Hyperscanning techniques, which measure inter-brain dynamics during naturalistic interactions, have further explored how interpersonal neural synchrony (INS; i.e., temporally coupled oscillatory activity between interacting individuals) indicates interaction quality. Previous studies demonstrate that prefrontal INS selectively predicts successful turn-taking and semantic alignment during natural conversations (Jiang et al. 2021, Zhang et al. 2021), whereas TPJ synchrony reflects shared attentional states and mental state inference (Liu et al. 2015, Zhang et al. 2017). These findings underscore the brain’s capacity to dynamically couple with social partners, forming a “neural dialogue”, beyond individual cognition (Hasson and Frith 2016).

Communication effectiveness is significantly influenced by its modality, with FTF and video-mediated communication (VMC) eliciting distinct neural and behavioural patterns. FTF, characterized by seamless integration of multimodal cues (e.g., gaze, prosody, gestures), enhances INS in the dorsolateral prefrontal cortex (dlPFC), a hub for real-time social cue integration and goal alignment (Jiang et al. 2021, Nguyen et al. 2021). Parent–child dyads exemplify this dynamic, exhibiting strengthened dlPFC coherence during FTF storytelling that correlates with emotional congruence (Schwartz et al. 2022). Conversely, VMC disrupts these dynamics by filtering nonverbal signals (e.g., peripheral gestures, haptic feedback) and introducing spatial-temporal delays, reducing INS in the dlPFC. Yet VMC is not uniformly disruptive. (Balters et al. 2023) reported that unfamiliar adults showed lower INS during socio-emotional dialogue on video, but higher INS during collaborative problem-solving, with further differences across frequency bands. Behavioural study has likewise shown that the benefits and costs of computer-mediated modality vary with relational intimacy, which refers to the degree of emotional closeness, mutual self-disclosure, and interdependence between partners. Specifically, closer partners interpret reduced-cue messages more accurately and repair breakdowns more efficiently (Caughlin and Sharabi 2013). Accordingly, examining how relational intimacy shapes interpersonal interaction across communication modalities can offer a more comprehensive understanding of communication in the digital era.

The Communicative Interdependence Perspective (CIP) can explain how intimacy affects neurobehavioral responses to communication constraints (Caughlin and Sharabi 2013, Tong and Walther 2015). According to CIP, FTF consolidate existing intimacy by enabling reciprocal adaptation to rich contextual cues, whereas VMC’s reduced cue richness may paradoxically benefit low-intimacy dyads by attenuating social evaluative pressure. For instance, strangers collaborating via VMC exhibit enhanced TPJ synchrony during problem-solving tasks, likely due to decreased anxiety and increased task focus (Balters et al. 2023). Despite these advances, previous studies have predominantly contrasted extreme groups (e.g., parents vs. strangers), leaving unresolved how gradations of relational intimacy modulate modality effects, particularly the distinction between romantic partnerships and friendships.

Among high-intimacy relationships, romantic partnerships represent a unique case due to their reliance on attachment-driven nonverbal synchrony (Feldman 2017). According to attachment theory, romantic relationships prioritize implicit coordination (e.g., gaze convergence, postural mimicry, and tactile contact) to maintain affective bonds (Hazan and Shaver 1994). Hyperscanning studies reveal that couples exhibit amplified INS in the anterior cingulate cortex (ACC) and dlPFC during FTF, patterns correlated with affective resonance and shared goal representation (Goldstein et al. 2018, Long et al. 2021). In contrast, friendships, lacking the attachment depth of romantic partnerships, adapt to VMC by prioritizing verbal reciprocity and task-focused collaboration (Reis et al. 2018, Balters et al. 2023). In sum, this divergence suggests that relational intimacy and relational typology jointly shape neurobehavioral synchrony.

Few studies have contrasted romantic couples with friends across more than one communication modality. Long et al. (2021) found that couples showed greater INS at 0.07–0.08 Hz during interpersonal touch, whereas opposite-sex friends showed stronger INS in the same band during verbal interaction, suggesting intimacy-dependent effects. Yet we still lack a systematic comparison of couples and friends in both FTF and VMC, and the frequency-specific mechanisms remain unclear. Moreover, prior fNIRS hyperscanning shows that interpersonal synchrony is not confined to a single “slow” range but clusters in several sub-bands below 0.30 Hz. Activity around 0.08*–*0.20 Hz increases during cooperation and mentalizing regions (Cui et al. 2012, Pan et al. 2021), whereas very-low frequencies 0.02*–*0.07 Hz rise with sustained interactive engagement and knowledge-related joint mental activity (Zheng et al. 2018). A broader 0.01*–*0.10 Hz window has also been linked to the multimodal cue integration and rapid turn-taking that characterize natural conversation (Jiang et al. 2012). These findings imply that faster sub-0.10 Hz rhythms support on-line cue exchange, whereas ultra-slow rhythms index prolonged joint monitoring. Addressing these gaps, we combine hyperscanning fNIRS with dyadic behaviour to test how intimacy and relationship type shape neurobehavioral synchrony in FTF and VMC.

This study employs a 2 × 2 mixed factorial design to explore how relational intimacy affects interpersonal synchrony across communication modalities. Neural activity was recorded using hyperscanning functional near-infrared spectroscopy (fNIRS), with optode arrays strategically positioned over the PFC and TPJ that regions central to social cognition (Koike et al. 2015, Jiang et al. 2021, Song et al. 2024). Dyads engage in naturalistic conversation, with verbal and non-verbal behaviours meticulously coded, and perform a paced joint button-press task both before and after each conversation, giving a domain-general motor baseline and outcome measure of interpersonal behavioural coordination and cooperative efficiency (Cui et al. 2012, Zhou et al. 2021). Combining pre/post button-press accuracy with verbal/non-verbal behaviours lets us quantify communication by integrating various indices, test its generalization across modalities, and relate every behavioural layer to frequency-specific INS. We hypothesize that couples will show stronger coordination and neural synchrony in FTF, while friends will exhibit enhanced synchrony in VMC.

## Materials and methods

### Participants

Seventy-two right-handed undergraduates (36 dyads; 50% female; mean age = 20.56 ± 1.62 years) were divided into two groups: (i) 18 opposite-sex friendship dyads (acquaintance duration 17.94 ± 10.24 months; relationship duration: 15.28 ± 7.03 months) and (ii) 18 opposite-sex romantic couples (acquaintance duration: 23.78 ± 16.79 months; relationship duration: 14.17 ± 11.24 months). Romantic relationships were validated using Sternberg’s Triangular Love Scale (intimacy ≥ 6.2, commitment ≥ 5.8, passion ≥ 6.0; Cronbach’s α  =  0.983) (Long et al. 2021). Independent samples *t*-tests confirmed group equivalence in age [*t* (70) = 0.56, *p *= 0.579, Cohen’s *d* = −0.14, 95% CI: −0.54, 1.00], acquaintance duration [*t* (34) = 1.259, *p *= 0.217, Cohen’s *d* = −0.41, 95% CI: −3.59, 15.25], or the duration of the established relationship [*t* (34) = −0.356, *p *= 0.724, Cohen’s *d* = 0.12, 95% CI: −7.46, 5.24]. Exclusion criteria included neurological/psychiatric history, communication disorders, and colour vision abnormalities (Ishihara test-verified). Written informed consent was received from all participants. The study protocol was approved by the Ethics Committee of Tianjin Normal University.

### Materials

#### Questionnaires

We characterized dyadic relationship with three well-validated instruments. Communication frequency was captured with a single 7-point Likert item (1 = “not at all,” 7 = “very frequently”; Caughlin and Sharabi 2013). Relationship satisfaction was measured with the 7-item Relationship Assessment Scale (1 = “low satisfaction,” 5 = “high satisfaction”; Hendrick 1988). Intimacy, passion, and commitment were assessed with the revised 36-item Triangular Love Scale (1 = “strongly disagree,” 9 = “strongly agree”; Long et al. 2023).

#### Evaluation of communication topics materials

Drawing on prior hyperscanning studies that used open-ended collaborative tasks (Jiang et al. 2012, Long et al. 2021, 2023), we curated six everyday planning topics to serve as naturalistic conversation: (i) collaborative planning of a camping trip; (ii) joint organization of an amusement park visit; (iii) cooperative coordination of a beach outing; (iv) mutual arrangement of mountain climbing/rock climbing activities; (v) collective scheduling of an aquarium visit; and (vi) collaborative preparation for a shopping trip. These topics were pretested by 120 independent raters (55 male, 65 female; mean age = 21.3 ± 2.1 years) who evaluated each topic’s hedonic valence on a 7-point scale. No significant gender differences were found in pleasure ratings for any topics. Finally, (1) and (2) were selected based on criteria emphasizing gender-neutral engagement, ecological validity, and exclusion of activities requiring specialized expertise (e.g., rock climbing techniques).

### Experimental procedure

**Figure 1.**
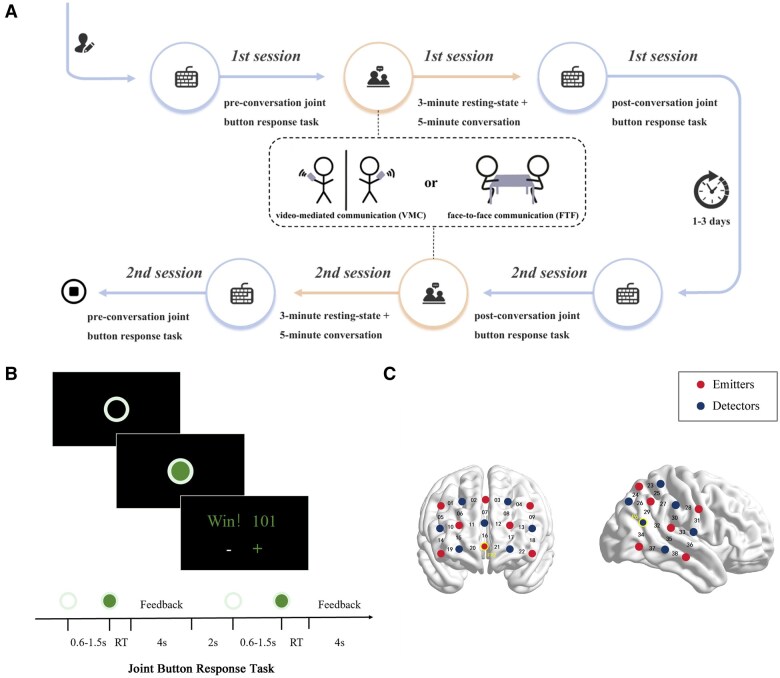
Experimental workflow and task paradigm. (A) Counter-balanced protocol: each dyad completed a face-to-face (FTF) and a video-mediated communication (VMC) session on separate days. Each session comprised a 3-min resting baseline, a 5-min naturalistic conversation on a pre-selected planning topic, and a paced button-press task administered both before and after the conversation. (B) after a variable fore-interval (0.6–1.5 s) the hollow grey circle turned green (go cue); partners pressed the Z (left) or/(right) key. A point was awarded when the inter-partner latency was ≤ 1∕8 of the combined reaction time. (C) fNIRS optode arrays covering the prefrontal cortex (PFC; 22 channels) and right temporo-parietal junction (TPJ; 16 channels) registered to the international 10–20 system.

The protocol (Fig. 1A) employed a counterbalanced, mixed design to assess modality and intimacy effects on interpersonal coordination. Each dyad completed two sessions (FTF and VMC) separated by a 24–72 hour washout period, with session order randomized. During each session, participants first underwent a 3-minute resting-state fNIRS recording. They then engaged in a 5-minute naturalistic conversation centred on a randomly assigned topics (amusement park visit or camping trip). Each dyad discussed one randomly assigned topic per session, with topic-modality combinations counterbalanced across participants to avoid confounding effects. The VMC condition mirrored FTF procedures using Tencent Meeting International Edition on iPad Pro 10.9-inch devices. All sessions were video-recorded. Before and after the conversation task, dyads performed two joint button tasks, with each comprising 20 trials. A single trial (Fig. 1B) began with a hollow gray circle (2.5°visual angle) displayed at screen centre. After a uniformly distributed fore-interval of 0.6-1.5s, the circle filled green, signalling “go”. Participant 1 responded with the “Z” key and the Participant 2 with the “/” key. If the partners’ RT difference was smaller than an adaptive synchrony threshold (see below), each earned one point; otherwise both lost a point. Dyads were instructed to maximize their joint score. After both participants pressed their response buttons, a 4 s feedback screen and 2 s inter-trial interval was shown.

### fNIRS data acquisition

The fNIRS data of both participants was simultaneously measured using two NIRSLAB devices with a sampling rate of 37 Hz (LABNIRS; Shimadzu Co., Japan). Following previous studies on social relationships (Long et al. 2021), a 3 × 5 optode array (8 emitters, 7 detectors, 22 channels) was placed over the prefrontal region, centred at Fpz and aligned with the Fp1-Fp2 line. Additionally, a 4 × 4 optode array (6 emitters, 6 detectors, 16 channels) was placed over the right TPJ, with the lowest probe aligned to P6 (Fig. 1C). These probe sets were checked and adjusted to ensure consistency across participants. Optodes were spaced 30 mm apart. The fNIRS channel positions were recorded using a 3D digitizer (FASTRAK, Polhemus, Colchester, VT, USA) and virtual registration method was used to determine the corresponded brain regions and MNI coordinates (Okamoto and Dan 2005, Singh et al. 2005; see Supplementary material).

### Data analysis

#### Behavioural data analysis

The joint button response task required dyads to synchronize their button presses in response to visual cues. On each trial:

*RT1:* Reaction time of Participant 1.

*RT2:* Reaction time of Participant 2.

##### Coordination threshold

A trial was considered synchronized if the absolute difference between RT1 and RT2 was less than (RT1 + RT2)/8, the thresholds was adapted from previous studies (Cui et al. 2012, Zhou et al. 2021) in order to control the difficulty of tasks at a reasonable level.

##### Coordination accuracy

This is the proportion of synchronized trials across all trials.


$$\mathrm{Accuracy}=\frac{\mathrm{Total}\mathrm{Number}\mathrm{of}\mathrm{Trial}}{\mathrm{Number}\mathrm{of}\mathrm{Synchronized}\mathrm{Trials}}\times 100\%$$

##### Accuracy difference

This quantifies the change in synchronization accuracy before vs. after conversation (FTF or VMC).


$$\text{Accuracy}\;\text{Difference}={\;\text{Accuracy}}_{\text{Post}-\text{conversation}}-{\text{Accuracy}}_{\text{Pre}-\text{conversation}}$$

A mixed-design ANOVA analysed accuracy differences in joint button response task using a 2 × 2 design: relationship type (couples, friends) as a between-subjects variable and communication modality (FTF, VMC) as a within-subjects variable. Simple effects were adjusted with the Bonferroni method.

Two coders who were unaware of the purpose of the experiment annotated dyads’ interactions from video recordings. *Verbal behaviour* was coded as the duration (unit: second) of speaking. *Nonverbal behaviour* was coded as the total duration (unit: second) of eye contact, laughter, head movement (such as nodding or head shaking), and gesture (hand/arm movements > 1 s). Inter-rater reliability, measured by intraclass correlation coefficients (ICC), showed strong consistency across all dyads (ICC = 0.82, 95% CI [0.76, 0.87]), meeting the standards for dyadic behavioural analysis as per previous studies (Long et al. 2023).

#### INS analysis

The raw optical signals underwent preprocessing: PCA removed global physiological noise by regressing out components with over 80% variance (Long et al. 2021). Motion artefacts were corrected using the CBSI algorithm (Cui et al. 2010, 2012). Data above 0.7 Hz and below 0.01 Hz were excluded to avoid high-frequency and very low-frequency noise and respiratory range data (0.15–0.3 Hz) were also removed (Guijt et al. 2007, Zheng et al. 2018). The first and last 30 seconds of each communication session were excluded to eliminate initiation and termination artefacts (Ding et al. 2014), resulting in 4 minutes of valid data.

Wavelet coherence (WTC) analysis was performed to calculate the INS (Cui et al. 2012). Thirty-eight measurement channels (22 prefrontal and 16 temporoparietal), resulting in 38 × 38 = 1444 CH combinations per dyad. Consistent with prior findings that neural coherence increases during social tasks relative to resting states (Cui et al. 2012, Jiang et al. 2012, Pan et al. 2021), we derived an INS increment metric by subtracting resting-state coherence from task-state coherence. The INS increment was calculated as:


$$\Delta \text{INS}=Z_{\mathrm{task}}-Z_{\mathrm{rest}}$$

where *Z*_task_ and *Z*_rest_ represent the normalized coherence during communication tasks and resting baseline, respectively. The following statistical tests were conducted on ΔINS.

The frequency cluster of interest was determined using a data-driven approach, following (Long et al. 2021). The above two-way mixed-design ANOVA (2 × 2) was conducted for each CH combination. The position of the frequency was determined by a statistically strict threshold at the *P* < .0005 to maximize sensitivity, whereas the width was determined by a relatively loose threshold at the *P* < .005 level. Multiple comparisons were corrected with a cluster-based permutation method (*P* < .05). For the permutation test, we adopted a pairwise shuffling approach to generate the null distribution of inter-brain synchronization. Specifically, we randomly reassigned dyad partners while preserving the original temporal structure of each participant’s hemodynamic signals (1000 iterations). This procedure effectively breaks any true inter-brain correlations while maintaining the intrinsic properties of individual fNIRS time series. For each shuffled pair, we recalculated the wavelet coherence and extracted cluster-level statistics identical to the real analysis. The resulting distribution of maximal cluster values under the null hypothesis was then used to determine the statistical significance of observed INS (two-tailed threshold at *P *< .05, cluster-defining threshold at *P *< .01). This conservative approach controls for Type I error while accounting for multiple comparisons across channels and frequency bands (Bilek et al. 2015). Based on this process, 2 frequency ranges of interest, that is, 0.081–0.09 Hz and 0.038–0.046 Hz. Finally, coherence values within these bands were averaged across time and subsequently analysed using a second 2 × 2 ANOVA.

To investigate the directional neural influences between dyad members, Granger causality analysis was implemented using the Multivariate Granger Causality (MVGC) toolbox (Bilek et al. 2015). This method evaluates how effectively one partner’s past neural activity predicts the other’s subsequent neural signals, operationalizing leader-follower dynamics in real-time interactions. The Granger causality index (GCI) was calculated by comparing prediction errors from two scenarios: (i) modelling a partner’s neural activity using only their own past data, and (ii) incorporating the other partner’s historical neural activity as an additional predictor.

## Results

### Behaviour coordination results

**Figure 2.**
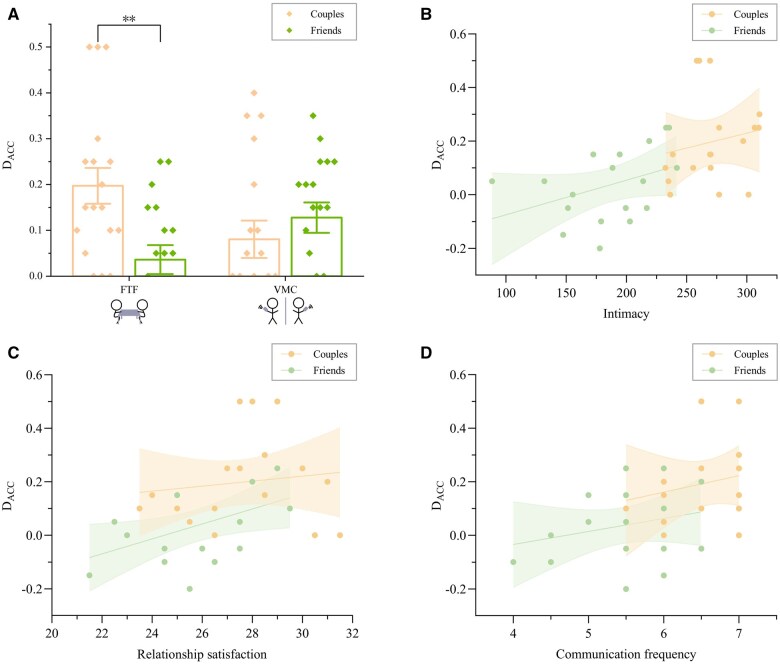
Joint button response task performance. (A) Accuracy difference (post- vs. pre-conversation) in the joint button response task across relationship type (couples vs. friends) and communication modalities (FTF vs. VMC). Couples gained most in FTF; friends gained most in VMC. (B–D) Pearson correlations between accuracy difference and (B) intimacy, (C) relationship satisfaction, and (D) communication frequency. Error bars denote ±SEM.

Behavioural coordination patterns significantly differed between romantic couples and friendship dyads across communication modalities. A 2(relationship type: couples, friends) × 2 (communication modality: FTF, VMC) mixed-design ANOVA was conducted on the Accuracy Difference in the joint button response task (Fig. 2A). The results showed a significant interaction effect, *F*(1, 34) = 8.88, *P *= .005, η^2^ = 0.21. Simple effects analysis revealed that during FTF communication, romantic couples showed significantly larger accuracy differences (M ± SD: 0.197 ± 0.166) than friends (0.036 ± 0.134), *F*(1, 34) = 10.30, *P *= .003, η^2^ = 0.23. In contrast, no significant difference was observed between couples (0.081 ± 0.037) and friends (0.128 ± 0.037) in VMC, *F*(1, 34) = 0.81, *P *= .376. Additionally, accuracy differences under FTF showed positive correlations with intimacy (*r *= 0.53, *P *= .001) (Fig. 2B), relationship satisfaction (*r *= 0.41, *P *= .014) (Fig. 2C) and communication frequency (*r *= 0.47, *P *= .004) (Fig. 2D), supporting the hypothesis that intimacy modulates modality-dependent behavioural synchrony.

### Verbal/nonverbal behavior results

A 2 (relationship type: couples vs. friends) × 2 (communication modality: FTF vs. VMC) mixed-design ANOVA was conducted to examine verbal and nonverbal behaviour patterns.

**Figure 3.**
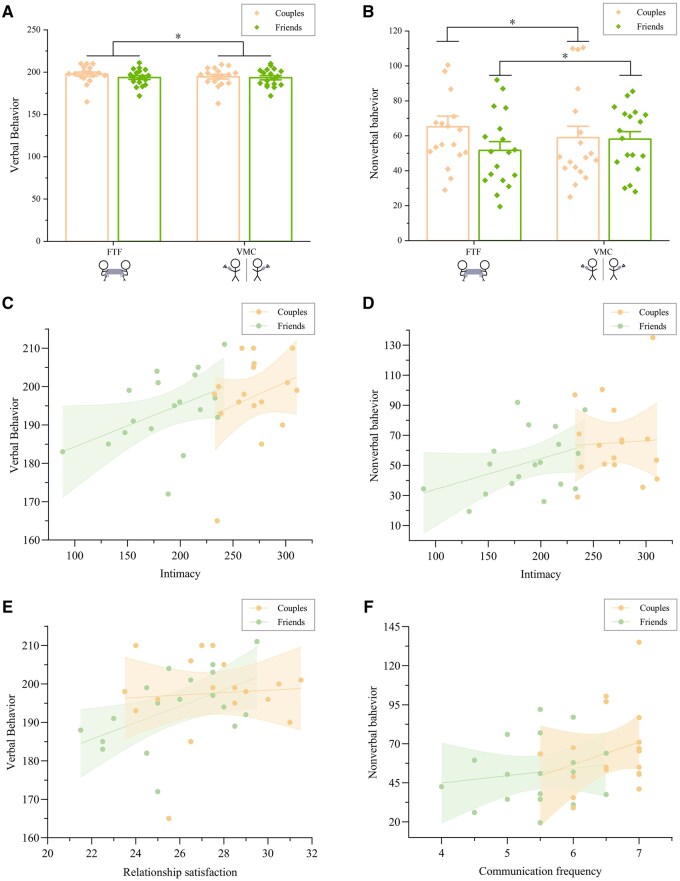
Verbal and nonverbal behaviours. (A) Verbal behaviours (conversational turns and total speaking time) across relationship type and modalities. (B) Nonverbal behaviours (eye contact duration, laughter duration, head movement duration, and gesture duration) showing an interaction. (C–F) Significant correlations during FTF: (C) verbal behaviour with intimacy; (D) nonverbal behaviour with intimacy; (E) verbal behaviour with relationship satisfaction; (F) non-verbal behaviour with communication frequency. Error bars denote ±SEM.

For verbal behaviour (Fig. 3A), the results displayed a significant main effect of communication modality, *F*(1, 34) = 4.59, *P *= .039, η^2^ = 0.12, with higher verbal behaviour scores in FTF (195.64 ± 10.19) than in VMC (194.00 ± 10.51). Neither the main effect of relationship type [*F*(1, 34) = 0.53, *P *= .47] nor the interaction [*F*(1, 34) = 3.17, *P *= .084] reached statistical significance.

For nonverbal behaviour (Fig. 3B), the analysis showed a significant interaction effect, *F*(1, 34) = 8.52, *P *= .006, η^2^ = 0.20. Simple effects analysis demonstrated that romantic couples exhibited stronger nonverbal behaviour during FTF communication (65.18 ± 25.941) compared to VMC (58.97 ± 27.567), *F*(1, 34) = 4.14, *P *= .05, η^2^ = 0.11. Conversely, friends showed enhanced nonverbal behaviour in VMC (58.08 ± 18.41) relative to FTF (51.69 ± 21.07), *F*(1, 34) = 4.36, *P *= .044, η^2^ = 0.11.

During FTF, behavioural coordination showed clear links to relationship measures. Verbal behaviour exhibited moderate positive correlations with intimacy (*r *= 0.38, *P *= .023; Fig. 3C) and relationship satisfaction (*r *= 0.33, *P *= .048; Fig. 3E). Nonverbal behaviour further demonstrated significant linkages with both intimacy (*r *= 0.36, *P *= .033; Fig. 3D) and communication frequency (*r *= 0.34, *P *= .045; Fig. 3F). These patterns support the view that verbal exchanges scaffold emotional disclosure, whereas embodied alignment reinforces affiliative bonds, yielding a behavioural index of relationship quality. No comparable associations emerged under VMC (see Figure S2).

### Neural synchronization results

**Figure 4.**
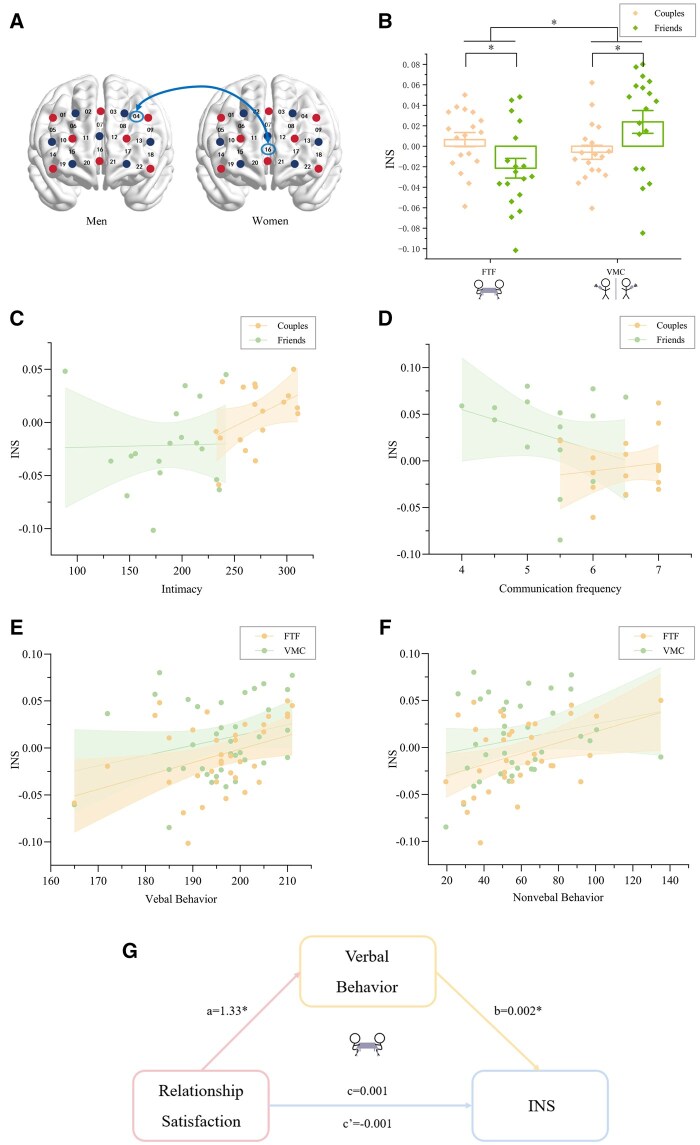
High-frequency INS (0.081–0.09 Hz) in the prefrontal cortex. (A) Significant channels: female frontopolar cortex (CH16) and male left dlPFC (CH4). (B) INS by relationship and modality: couples > friends in FTF; friends > couples in VMC. (C–F) Correlations of INS with (C) intimacy, (D) verbal behaviour, (E) non-verbal behaviour and (F) communication frequency. (G) Mediation model: verbal behaviour fully mediated the effect of relationship satisfaction on high-frequency INS during FTF (95% CI = 0.002–0.0045). Error bars denote ±SEM.

For the 0.081–0.09 Hz band, the neural synchronization localized in the left dorsolateral PFC of males (CH4) and the frontal pole of females (CH16) (Fig. 4A). The ANOVA results showed a significant main effect of communication modality, *F*(1, 34) = 6.79, *P *= .014, η^2^ = 0.17, with neural synchronization values in FTF (−0.008 ± 0.037) significantly lower than in VMC (0.009 ± 0.041). A significant interaction [*F*(1, 34) = 20.86, *P *< .001, η^2^ = 0.38] showed romantic couples (0.007 ± 0.029) exhibited stronger prefrontal INS during FTF communication compared to friends (−0.022 ± 0.041), *F*(1, 34) = 5.61, *P *= .024, η^2^ = 0.14, whereas friends (0.023 ± 0.047) displayed higher synchrony in VMC than couples (−0.005 ± 0.029), *F*(1, 34) = 5.17, *P* = .029, η^2^ = 0.13 (Fig. 4C).

**Figure 5.**
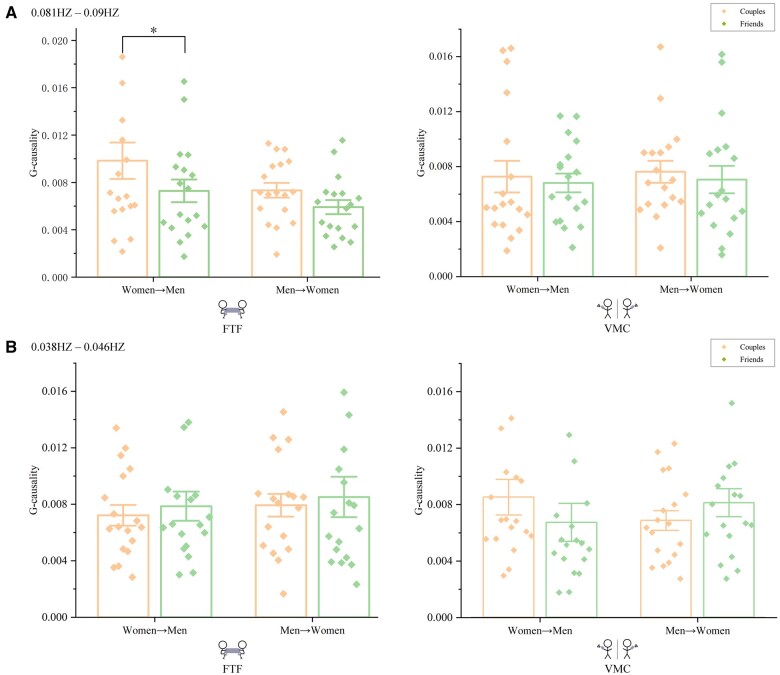
Directional influence assessed by Granger causality. (A) High-frequency (0.081–0.090 Hz) Granger-causality index (GCI) in FTF: female → male paths exceeded male → female. (B) Same band in VMC: no directional asymmetry. (C–D) Low-frequency (0.038–0.046 Hz) GCI showed no significant directional effects in either modality. Error bars denote ±SEM.

Granger causality analysis was conducted on the time series of female frontal pole (CH16) and male left dlPFC (CH4). For both FTF and VMC tasks, all directional influences (female→male, male→female) for both couples and friends were significantly greater than zero, *ps* < 0.05, confirming bidirectional neural coupling across modalities. A 2 (relationship type: couples, friends) × 2 (direction: female→male, male→female) mixed-design ANOVA was conducted separately for FTF and VMC tasks. For FTF communication (Fig. 5A), a significant main effect of direction [*F*(1, 34) = 4.27, *P *= .046, η^2^ = 0.11] showed that the female→male influence (0.009 ± 0.006) was higher than male→female influence (0.007 ± 0.003). Neither the main effect of relationship type, *F*(1, 34) = 3.48, *P *= .071, nor the interaction effect, *F*(1, 34) = 0.36, *P *= .553, reached significance. This female→male dlPFC influence during FTF aligns with neuroimaging evidence showing women’s greater engagement of prefrontal regions during socioemotional tasks (Christov-Moore et al. 2014), potentially reflecting enhanced top-down regulation of interpersonal alignment. Such gender differences in neural processing may stem from social-cognitive specialization (Shamay-Tsoory et al. 2019) rather than purely biological factors. In contrast, VMC task (Fig. 5B) showed no significant directional effects, *F*(1, 34) = 0.09, *P *= .763, relationship type, *F*(1, 34) = 0.34, *P *= .562, nor the interaction effect, *F*(1, 34) = 0.003, *P *= .957, indicating that there was no leader-follower dynamic present in VMC.

**Figure 6.**
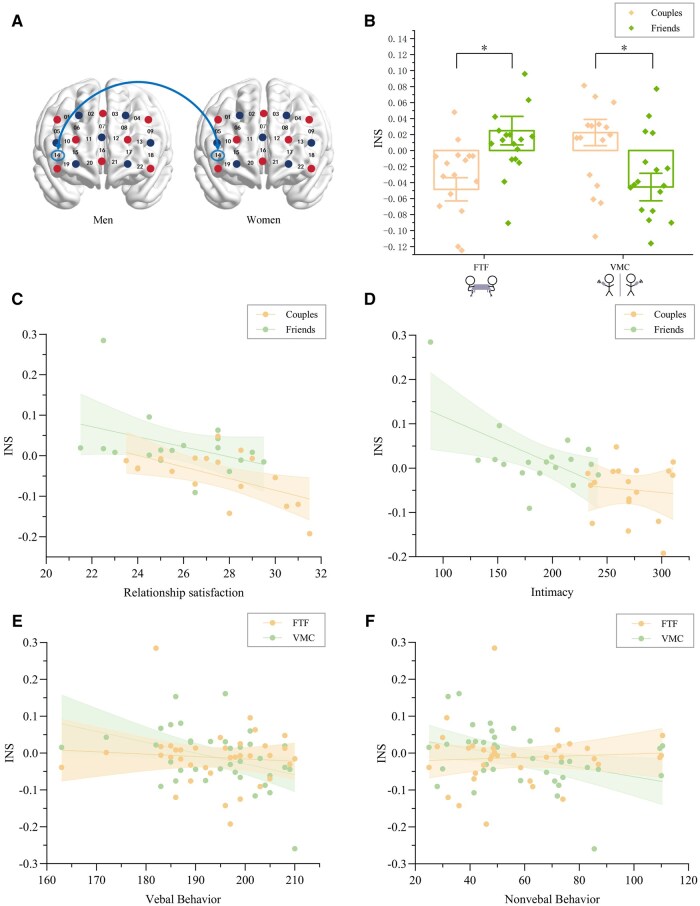
Low-frequency INS (0.038–0.046 Hz) in the right dlPFC. (A) Significant channel cluster centred on CH14 in both partners. (B) INS by relationship and modality: friends > couples in FTF; couples > friends in VMC. (C–F) Negative correlations of INS with (C) relationship satisfaction, (D) intimacy, (E) verbal behaviour, and (F) nonverbal behaviour.

For the 0.038–0.046 Hz band, the neural synchronization localized in right dorsolateral PFCx for both males and females (CH14) (Fig. 6A). The ANOVA results (Fig. 6B) showed a significant interaction effect, *F*(1, 34) = 20.58, *P *< .001, η^2^ = 0.38. Simple effects analysis found that friends (0.025 ± 0.076) exhibited stronger prefrontal INS during FTF relative to romantic couples (−0.049 ± 0.062), *F*(1, 34) = 10.14, *P *= .003, η^2^ = 0.23, whereas couples (0.022 ± 0.07) displayed higher synchrony in VMC than friends (−0.046 ± 0.073), *F*(1, 34) = 8.19, *P *= .007, η^2^ = 0.19, contrary to the results of 0.081–0.09 Hz Band.

Granger causality analysis was performed on hemodynamic series of female CH14 and male CH14. For both FTF and VMC tasks, all directional influences (female→male, male→female) for both couples and friends were significantly greater than zero, *ps* < 0.05, confirming bidirectional neural coupling across modalities. However, the results of a mixed-design ANOVA found no significant effect in FTF (Fig. 5C) (main effect of direction, *F*(1, 34) = 0.84, *P *= .367; main effect of relationship type, *F*(1, 34) = 0.24, *P *= .63; the interaction effect, *F*(1, 34) = 0.001, *P *= .97) and VMC condition (Fig. 5D) (main effect of direction, *F*(1, 34) = 0.02, *P *= .895; main effect of relationship type, *F*(1, 34) = 0.05, *P *= .825; the interaction effect, *F*(1, 34) = 2.29, *P *= .139), indicating that there was no leader-follower dynamic in either FTF or VMC contexts.

### Integration of behavioral and neural coordination

For the 0.081–0.09 Hz band, correlation analysis showed that during FTF, prefrontal INS exhibited significant positive correlations with intimacy (*r *= 0.39, *P *= .020) (Fig. 4C), verbal behaviour (*r *= 0.39, *P *= .020) (Fig. 4D), and nonverbal behaviour (*r *= 0.38, *P *= .024) (Fig. 4E). In VMC, a significant negative correlation with INS and communication frequency (*r *=* −*0.37, *P *= .026) (Fig. 4D), and similar positive correlations with verbal (*r *= 0.42, *P *= .011) (Fig. 4E) and nonverbal behaviour (*r *= 0.39, *P *= .020) (Fig. 4F). Mediation analysis further demonstrated that verbal behaviour fully mediated the effect of relationship satisfaction on prefrontal INS during FTF communication (lower confidence interval = 0.002, upper confidence interval = 0.0045) (Fig. 4G).

For the 0.038–0.046 Hz band, correlation analysis showed that during FTF, INS exhibited significant negative correlations with relationship satisfaction (*r *=* −*0.55, *P *= .001) (Fig. 6C), and intimacy (*r *=* −*0.59, *P *< .001) (Fig. 6D). In VMC, similar associations emerged between INS and verbal (*r *=* −*0.39, *P *= .017) (Fig. 6E) and nonverbal behaviour (*r *=* −*0.37, *P *= .026) (Fig. 6F).

## Discussion

The present study demonstrates that the impact of communication modality on INS is jointly sculpted by relationship intimacy and neural frequency. In the high-frequency band (0.081–0.090 Hz), synchrony was higher for romantic couples during face-to-face conversation (FTF) and for friends during VMC, whereas the low-frequency band (0.038–0.046 Hz) showed the opposite pattern. This dual crossover positions intimacy as a contextual gatekeeper that determines which temporal channel the brain recruits under a given communication modality. By integrating these findings, we advance a relational-context model, reconciling prior heterogeneous results on VMC and broadens our understanding of social interaction in the digital era: the neural coordination is dynamically reconfigured by the interplay between technologically mediated channels and partners’ relational history.

### A relational-context model for intimacy-by-modality interaction

Attachment theory helps explain the couples’ advantage in FTF. Affective security in romantic bonds is maintained largely through implicit sensorimotor attunement (e.g., subtle gaze convergence, postural alignment, and touch), FTF preserves the very cues that couples habitually exploit, thereby maximizing their neural alignment. The CIP (Caughlin and Sharabi 2013, Tong and Walther 2015) complements this account for friends. Friendships rely more on explicit verbal scaffolding and shared task talk, resources that remain largely intact in VMC, allowing friends to surpass couples when bodily cues are sparse.

However, previous theories remain unclear on how the frequency-specific neural rhythms implements this intimacy-by-modality effects. In the 0.081*–*0.090 Hz band, INS was largest when each dyad could draw on its primary fast-cue channel: couples in FTF, where touch, gaze, and posture are fully available, and friends in VMC, where clear audio and a fixed frontal camera preserve the explicit speech cues and prosodic contours on which friendships typically rely for coordination. This arrangement gives each relationship type ready access to the moment-to-moment signals it habitually exploits. This fits previous fNIRS evidence that activity near 0.08 Hz tracks on-line cue integration: (Jiang et al. 2012) showed that power in this range scales with rapid turn-taking, and (Cui et al. 2012) linked it to executive control over social signals. Thus, when partners have rich embodied cues (couples-FTF) or intact verbal scaffolding (friends-VMC), high-frequency synchrony is up-regulated to fuse those instantaneous signals. Conversely, in the slower 0.038*–*0.046 Hz band, INS tightened when the preferred fast-cue stream was constrained: friends in FTF, who must monitor a dense but less familiar set of embodied signals, and couples in VMC, who strive to maintain intimacy through impoverished sensory input. This pattern accords with evidence that sub-0.05 Hz fluctuations rise during sustained interactive engagement (Zheng et al. 2018).

Therefore, we synthesize these findings in a relational-context model that extends previous theories in three ways. First, relationship sets the initial weighting of fast- versus slow-frequency synchrony; communication modality modulates the availability of cues that feed those bands. Second, when a primary channel is degraded (e.g., embodied cues in VMC for couples), coupling migrates to the alternative band that best supports the remaining information (slow socio-emotional monitoring). Third, the changes in verbal alignment, non-verbal mirroring, and pre/post motor synchrony parallel the neural shifts, indicating a coherent multilevel adaptation. Recent findings align with this framework. Balters et al. (2023) showed that unfamiliar partners in VMC exhibited reduced INS during socio-emotional exchange yet enhanced INS during problem-solving, precisely the pattern expected when intimacy is low and task demands privilege either fast or slow cues. Long et al. (2021) found that couples outperformed friends in a tactile task but not in a vocal one, consistent with our claim that the advantage conferred by intimacy appears only when the relevant cue channel is available.

### Neurobehavioral compensation via verbal precision and nonverbal attenuation

Intimacy-dependent communication modalities elicit divergent neurobehavioral strategies through verbal precision and nonverbal attenuation. For romantic couples, verbal behaviour fully mediated the relationship between relationship satisfaction and high-frequency dlPFC synchrony during FTF, reflecting their reliance on affective language and conversational reciprocity to reinforce shared goal representation. This verbal-driven coordination aligns with the role of the dlPFC in integrating multimodal cues (e.g., prosody, gaze) to sustain emotional attunement through predictive coding mechanisms (Feldman 2017, Long et al. 2021). Conversely, friends engaging in VMC exhibited compensatory verbal precision (e.g., explicit role allocation, logical sequencing), reflecting a mechanism supporting sustained task monitoring and explicit goal alignment through cognitive scaffolding (Zheng et al. 2020). Notably, while couples attenuated nonverbal reciprocity in VMC to conserve cognitive resources, friends amplified verbal precision to counteract the reduced ecological validity of nonverbal channels, revealing an intimacy-modulated trade-off between neural alignment efficiency and behavioural synchrony costs.

Unlike precise verbal strategies, nonverbal behaviour revealed an intimacy-modulated dissociation in neural alignment efficiency. Couples sustained nonverbal synchrony (e.g., gaze convergence, postural mimicry) during FTF, reflecting a mechanism facilitating dyad-specific sensorimotor resonance (Feldman 2017). However, in VMC, their gesture kinematics and microexpression synchrony decrease. These contrasts align with friends’ compensatory amplification of visually salient nonverbal signals (e.g., exaggerated head movements, hyperarticulated prosody) in VMC (Zheng et al. 2020).

### Directional neural asymmetry via granger causality

In romantic couples, Granger-causal analysis showed that neural activity in the female partner’s frontal pole (CH16) consistently predicted subsequent activity in the male partner’s dlPFC (CH4) during FTF. This female→male influence mirrors meta-analytic evidence that women recruit the mirror-empathy network more strongly and decode micro-expressions with higher fidelity (Christov-Moore et al. 2014), and accords with findings that females show greater dlPFC engagement in socio-emotional tasks (Shamay-Tsoory et al. 2019). Rather than implying immutable evolutionary roles (Fisher et al. 2016), the asymmetry likely reflects gender-linked socialization and hormone-mediated tuning of affective-prediction circuits, which can be attenuated by contextual constraints. A parallel attenuation has been reported for mother-child dyads: maternal leading of the child’s neural activity is robust in FTF but significantly reduced online (Schwartz et al. 2022), suggesting that VMC generally weaken sensitive-linked predictive coupling.

### Contribution and future directions

Several constraints temper these conclusions. First, the sample comprised hetero-romantic couples and same-sex friendships drawn from a single cultural milieu; broader relationship types and cultural contexts may reveal different coupling rules. Second, only two slow hemodynamic bands were analysed; finer spectral bins or complementary modalities (e.g., EEG) could uncover additional timescales of adaptation. Third, our VMC condition used a conventional webcam interface without haptic or spatial-audio enrichment; future work should test whether higher-fidelity platforms mitigate the observed decrements. Finally, Granger causality was restricted to dlPFC and frontopolar channels; whole-cortex connectivity might expose further directional pathways.

Future studies can reimagine of interaction technologies. Platforms preserving high-frequency nonverbal cues, such as high-fidelity gesture tracking or real-time physiological synchrony displays, could mitigate VMC’s erosive effects on intimate dyads. AI-driven interfaces might dynamically adapt design elements based on detected relational intimacy, prioritizing verbal clarity for strangers and nonverbal richness for couples. By bridging social neuroscience with human–computer interaction, such innovations could transform digital communication from a compromise into a context-aware enhancement of human connection.

## Supplementary Material

nsaf070_Supplementary_Data

## Data Availability

The data and code underlying this article will be shared on reasonable request to the corresponding author.

## References

[nsaf070-B1] Balters S , Miller JG, Li R et al Virtual (zoom) interactions alter conversational behavior and interbrain coherence. J Neurosci 2023;43:2568–78. 10.1523/JNEUROSCI.1401-22.2023 36868852 PMC10082458

[nsaf070-B2] Bilek E , Ruf M, Schäfer A et al Information flow between interacting human brains: Identification, validation, and relationship to social expertise. Proc Natl Acad Sci USA 2015;112:5207–12. 10.1073/pnas.1421831112 25848050 PMC4413334

[nsaf070-B3] Caughlin J.P. , Sharabi LL. A communicative interdependence perspective of close relationships: the connections between mediated and unmediated interactions matter. J Commun 2013;63:873–93. 10.1111/jcom.12046

[nsaf070-B4] Christov-Moore L , Simpson EA, Coudé G et al Empathy: Gender effects in brain and behavior. Neurosci Biobehav Rev 2014;46:604–27. 10.1016/j.neubiorev.2014.09.001 25236781 PMC5110041

[nsaf070-B5] Cui X , Bray S, Reiss AL. Functional near infrared spectroscopy (NIRS) signal improvement based on negative correlation between oxygenated and deoxygenated hemoglobin dynamics. NeuroImage 2010;49:3039–46. 10.1016/j.neuroimage.2009.11.050 19945536 PMC2818571

[nsaf070-B6] Cui X , Bryant DM, Reiss AL. NIRS-based hyperscanning reveals increased interpersonal coherence in superior frontal cortex during cooperation. NeuroImage 2012;59:2430–7. 10.1016/j.neuroimage.2011.09.003 21933717 PMC3254802

[nsaf070-B7] Ding XP , Sai L, Fu G et al Neural correlates of second-order verbal deception: a functional near-infrared spectroscopy (fNIRS) study. NeuroImage 2014;87:505–14. 10.1016/j.neuroimage.2013.10.023 24161626

[nsaf070-B8] Feldman R. The neurobiology of human attachments. Trends Cogn Sci 2017;21:80–99. 10.1016/j.tics.2016.11.007 28041836

[nsaf070-B9] Fisher HE , Xu X, Aron A et al Intense, passionate, romantic love: a natural addiction? How the fields that investigate romance and substance abuse can inform each other. Front Psychol 2016;7:1–10. 10.3389/fpsyg.2016.00687 27242601 PMC4861725

[nsaf070-B10] Frith CD , Frith U. The neural basis of mentalizing. Neuron 2006;50:531–4. 10.1016/j.neuron.2006.05.001 16701204

[nsaf070-B11] Goldstein P , Weissman-Fogel I, Dumas G et al Brain-to-brain coupling during handholding is associated with pain reduction. Proc Natl Acad Sci USA 2018;115:E2528–37. 10.1073/pnas.1703643115 29483250 PMC5856497

[nsaf070-B12] Guijt AM , Sluiter JK, Frings-Dresen MHW. Test-retest reliability of heart rate variability and respiration rate at rest and during light physical activity in normal subjects. Arch Med Res 2007;38:113–20. 10.1016/j.arcmed.2006.07.009 17174734

[nsaf070-B13] Hari R , Henriksson L, Malinen S et al Centrality of social interaction in human brain function. Neuron 2015;88:181–93. 10.1016/j.neuron.2015.09.022 26447580

[nsaf070-B14] Hasson U , Frith CD. Mirroring and beyond: Coupled dynamics as a generalized framework for modelling social interactions. Philos Trans R Soc B 2016;371:20150366. 10.1098/rstb.2015.0366 PMC4843605 27069044

[nsaf070-B15] Hasson U , Ghazanfar AA, Galantucci B et al Brain-to-brain coupling: a mechanism for creating and sharing a social world. Trends Cogn Sci 2012;16:114–21. 10.1016/j.tics.2011.12.007 22221820 PMC3269540

[nsaf070-B16] Hazan C , Shaver PR. Attachment as an organizational framework for research on close relationships. Psychol Inq 1994;5:1–22. 10.1207/s15327965pli0501_1

[nsaf070-B17] Hendrick SS. A generic measure of relationship satisfaction. J Marriage Family 1988;50:93–8. 10.2307/352430

[nsaf070-B18] Jiang J , Dai B, Peng D et al Neural synchronization during face-to-face communication. J Neurosci 2012;32:16064–9. 10.1523/JNEUROSCI.2926-12.2012 23136442 PMC6621612

[nsaf070-B19] Jiang J , Zheng L, Lu C. A hierarchical model for interpersonal verbal communication. Soc Cogn Affect Neurosci 2021;16:246–55. 10.1093/scan/nsaa151 33150951 PMC7812628

[nsaf070-B20] Koike T , Tanabe HC, Sadato N. Hyperscanning neuroimaging technique to reveal the ‘two-in-one’ system in social interactions. Neurosci Res 2015;90:25–32. 10.1016/j.neures.2014.11.006 25499683

[nsaf070-B21] Liu T , Saito H, Oi M. Role of the right inferior frontal gyrus in turn-based cooperation and competition: a near-infrared spectroscopy study. Brain Cogn 2015;99:17–23. 10.1016/j.bandc.2015.07.001 26189111

[nsaf070-B22] Long Y , Zheng L, Zhao H et al Interpersonal neural synchronization during interpersonal touch underlies affiliative pair bonding between romantic couples. Cereb Cortex 2021;31:1647–59. 10.1093/cercor/bhaa316 33145593

[nsaf070-B23] Long Y , Zhong M, Aili R et al Transcranial direct current stimulation of the right anterior temporal lobe changes interpersonal neural synchronization and shared mental processes. Brain Stimul 2023;16:28–39. 10.1016/j.brs.2022.12.009 36572209

[nsaf070-B24] Nguyen T , Schleihauf H, Kayhan E et al Neural synchrony in mother-child conversation: Exploring the role of conversation patterns. Soc Cogn Affect Neurosci 2021;16:93–102. 10.1093/scan/nsaa079 32591781 PMC7812624

[nsaf070-B25] Okamoto M , Dan I. Automated cortical projection of head-surface locations for transcranial functional brain mapping. NeuroImage 2005;26:18–28. 10.1016/j.neuroimage.2005.01.018 15862201

[nsaf070-B26] Pan Y , Guyon C, Borragán G et al Interpersonal brain synchronization with instructor compensates for learner’s sleep deprivation in interactive learning. Biochem Pharmacol 2021;191:114111. 10.1016/j.bcp.2020.114111 32569629

[nsaf070-B27] Redcay E , Schilbach L. Using second-person neuroscience to elucidate the mechanisms of social interaction. Nat Rev Neurosci 2019;20:495–505. 10.1038/s41583-019-0179-4 31138910 PMC6997943

[nsaf070-B28] Reis HT , Lee KY, O’Keefe SD et al Perceived partner responsiveness promotes intellectual humility. J Exp Soc Psychol 2018;79:21–33. 10.1016/j.jesp.2018.05.006

[nsaf070-B29] Schwartz L , Levy J, Endevelt-Shapira Y et al Technologically-assisted communication attenuates inter-brain synchrony. NeuroImage 2022;264:119677. 10.1016/j.neuroimage.2022.119677 36244598

[nsaf070-B30] Shamay-Tsoory SG , Saporta N, Marton-Alper IZ et al Herding brains: a core neural mechanism for social alignment. Trends Cogn Sci 2019;23:174–86. 10.1016/j.tics.2019.01.002 30679099

[nsaf070-B31] Singh AK , Okamoto M, Dan H et al Spatial registration of multichannel multi-subject fNIRS data to MNI space without MRI. NeuroImage 2005;27:842–51. 10.1016/j.neuroimage.2005.05.019 15979346

[nsaf070-B32] Song X , Dong M, Feng K et al Influence of interpersonal distance on collaborative performance in the joint simon task—an fNIRS-based hyperscanning study. NeuroImage 2024;285:120473. 10.1016/j.neuroimage.2023.120473 38040400

[nsaf070-B33] Tong ST , Walther JB. The confirmation and disconfirmation of expectancies in computer-mediated communication. Commun Res 2015;42:186–212.

[nsaf070-B34] Zhang M , Jia H, Zheng M et al Group decision-making behavior in social dilemmas: inter-brain synchrony and the predictive role of personality traits. Personal Individ Differ 2021;168:110315. 10.1016/j.paid.2020.110315

[nsaf070-B35] Zhang M , Liu T, Pelowski M et al Social risky decision-making reveals gender differences in the TPJ: a hyperscanning study using functional near-infrared spectroscopy. Brain Cogn 2017;119:54–63. 10.1016/j.bandc.2017.08.008 28889923

[nsaf070-B37] Zheng L , Chen C, Liu W et al Enhancement of teaching outcome through neural prediction of the students’ knowledge state. Human Brain Mapping 2018;39:3046–57. 10.1002/hbm.24059 29575392 PMC6866636

[nsaf070-B38] Zheng L , Liu W, Long Y et al Affiliative bonding between teachers and students through interpersonal synchronisation in brain activity. Soc Cogn Affect Neurosci 2020;15:97–109. 10.1093/scan/nsaa016 32022237 PMC7171379

[nsaf070-B39] Zhou X , Pan Y, Zhang R et al Mortality threat mitigates interpersonal competition: an EEG-based hyperscanning study. Soc Cogn Affect Neurosci 2021;16:621–31. 10.1093/scan/nsab033 33755182 PMC8138089

