# Supplementary material for: Love in the time of Zoom: how intimacy modulates brain and behaviour synchrony in face-to-face versus video communication
Source: Soc Cogn Affect Neurosci. 2025 Jul 10;20(1):nsaf070. doi: 10.1093/scan/nsaf070 (PMC12413897; doi:10.1093/scan/nsaf070)
Supplement: nsaf070_Supplementary_Data [file nsaf070_supplementary_data.docx]

**Supplementary material**

Table S1. The Montreal Neurological Institute (MNI) brain spacing of fNIRS channels.

| Channel | MNI | | | Brodmann area | Probability (%) |
| --- | --- | --- | --- | --- | --- |
|  | X | Y | Z |  |  |
| CH01 | 36 | 40 | 44 | 9 - Dorsolateral prefrontal cortex | 0.894 |
| CH02 | 15 | 56 | 43 | 9 - Dorsolateral prefrontal cortex | 0.996 |
| CH03 | -12 | 58 | 41 | 9 - Dorsolateral prefrontal cortex | 0.939 |
| CH04 | -35 | 45 | 39 | 9 - Dorsolateral prefrontal cortex | 0.516 |
| CH05 | 48 | 37 | 36 | 45 - pars triangularis Broca's area | 0.581 |
| CH06 | 28 | 59 | 31 | 46 - Dorsolateral prefrontal cortex | 0.561 |
| CH07 | 5 | 64 | 32 | 10 - Frontopolar area | 0.762 |
| CH08 | -23 | 62 | 29 | 10 - Frontopolar area | 0.46 |
| CH09 | -44 | 44 | 28 | 45 - pars triangularis Broca's area | 0.635 |
| CH10 | 40 | 57 | 23 | 46 - Dorsolateral prefrontal cortex | 0.878 |
| CH11 | 18 | 70 | 21 | 10 - Frontopolar area | 1 |
| CH12 | -13 | 70 | 20 | 10 - Frontopolar area | 1 |
| CH13 | -35 | 61 | 18 | 46 - Dorsolateral prefrontal cortex | 0.569 |
| CH14 | 50 | 50 | 12 | 46 - Dorsolateral prefrontal cortex | 0.678 |
| CH15 | 30 | 68 | 11 | 10 - Frontopolar area | 0.91 |
| CH16 | 8 | 73 | 11 | 10 - Frontopolar area | 1 |
| CH17 | -23 | 70 | 9 | 10 - Frontopolar area | 0.905 |
| CH18 | -45 | 54 | 6 | 46 - Dorsolateral prefrontal cortex | 0.822 |
| CH19 | 42 | 62 | -1 | 10 - Frontopolar area | 0.615 |
| CH20 | 18 | 73 | -1 | 10 - Frontopolar area | 0.542 |
| CH21 | -13 | 73 | -2 | 10 - Frontopolar area | 0.571 |
| CH22 | -36 | 64 | -4 | 10 - Frontopolar area | 0.504 |
| CH23 | 21 | -75 | 61 | 7 - Somatosensory Association Cortex | 1 |
| CH24 | 23 | -85 | 49 | 19 - V3 | 0.588 |
| CH25 | 36 | -68 | 59 | 7 - Somatosensory Association Cortex | 0.968 |
| CH26 | 38 | -79 | 45 | 19 - V3 | 0.509 |
| CH27 | 52 | -59 | 54 | 40 - Supramarginal gyrus part of Wernicke's area | 0.542 |
| CH28 | 55 | -31 | 58 | 1 - Primary Somatosensory Cortex | 0.462 |
| CH29 | 51 | -71 | 42 | 39 - Angular gyrus, part of Wernicke's area | 0.949 |
| CH30 | 62 | -45 | 48 | 40 - Supramarginal gyrus part of Wernicke's area | 1 |
| CH31 | 62 | -18 | 50 | 1 - Primary Somatosensory Cortex | 0.432 |
| CH32 | 61 | -59 | 35 | 39 - Angular gyrus, part of Wernicke's area | 0.703 |
| CH33 | 69 | -32 | 39 | 40 - Supramarginal gyrus part of Wernicke's area | 0.595 |
| CH34 | 56 | -73 | 19 | 39 - Angular gyrus, part of Wernicke's area | 0.628 |
| CH35 | 68 | -47 | 23 | 22 - Superior Temporal Gyrus | 0.724 |
| CH36 | 70 | -17 | 26 | 2 - Primary Somatosensory Cortex | 0.483 |
| CH37 | 65 | -60 | 6 | 37 - Fusiform gyrus | 0.864 |
| CH38 | 72 | -37 | 7 | 22 - Superior Temporal Gyrus | 0.825 |


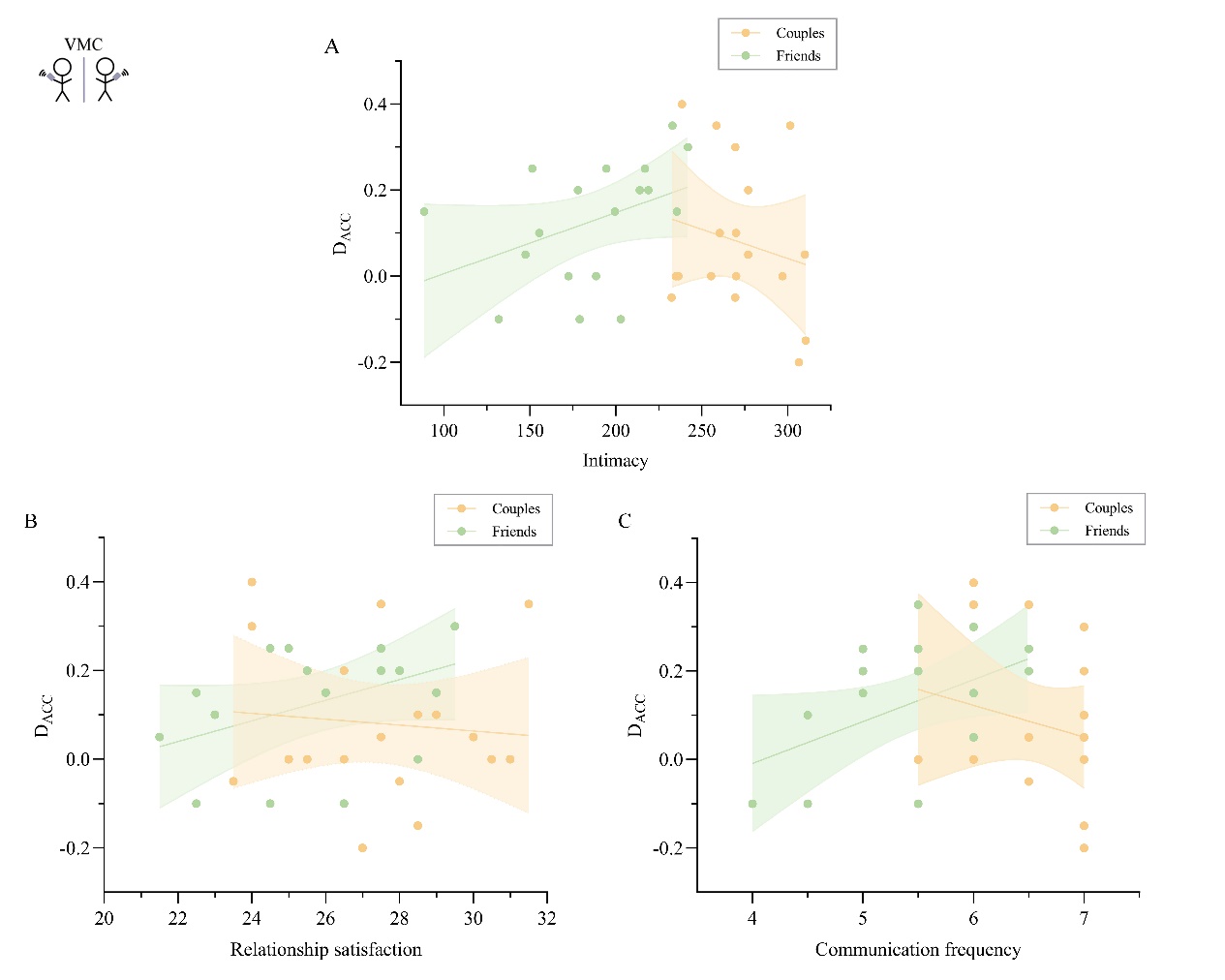
The supplementary analyses examine the relationships among behavioral coordination, frequency-specific interpersonal neural synchrony (INS), and intimacy-related questionnaires in both face-to-face communication (FTF) and video-mediated communication (VMC).

**Figure S1 Behavioral coordination during VMC**

Pearson correlations between accuracy difference (post- vs. pre-conversation) and (A) intimacy (*r* = -0.04, *p* = .807), (B) relationship satisfaction (*r* = 0.07, *p* = .706), and (C) communication frequency (*r* = -0.01, *p* = .972). No associations were significant.


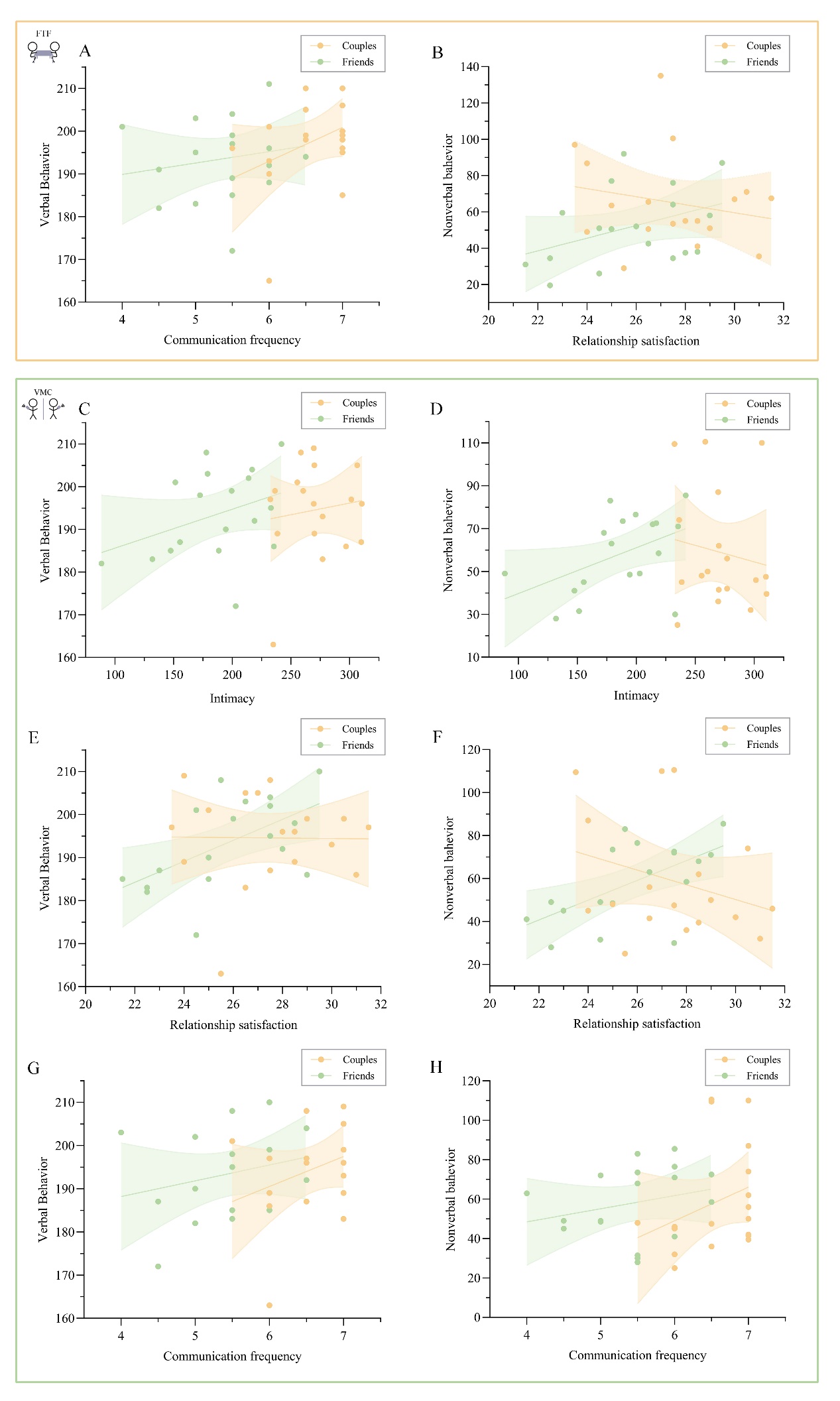


Figure S2 Verbal and non-verbal coordination in FTF and VMC

**During FTF: (A)** Verbal coordination versus communication frequency (*r* = 0.32, *p* = .059). **(B)** Non-verbal coordination versus relationship satisfaction (*r* = 0.15, *p* = .384).

**During VMC:** Verbal coordination with **(C)** intimacy (*r* = 0.20, *p* = .244), **(E)** relationship satisfaction (*r* = 0.27, *p* = .112), and **(G)** communication frequency *(r* = 0.23, *p* = .182). Non-verbal coordination with **(D)** intimacy (*r* = 0.11, *p* = .529), **(F)** relationship satisfaction (*r* = 0.06, *p* = .736), and **(H)** communication frequency (*r* = 0.20, *p* = .245).


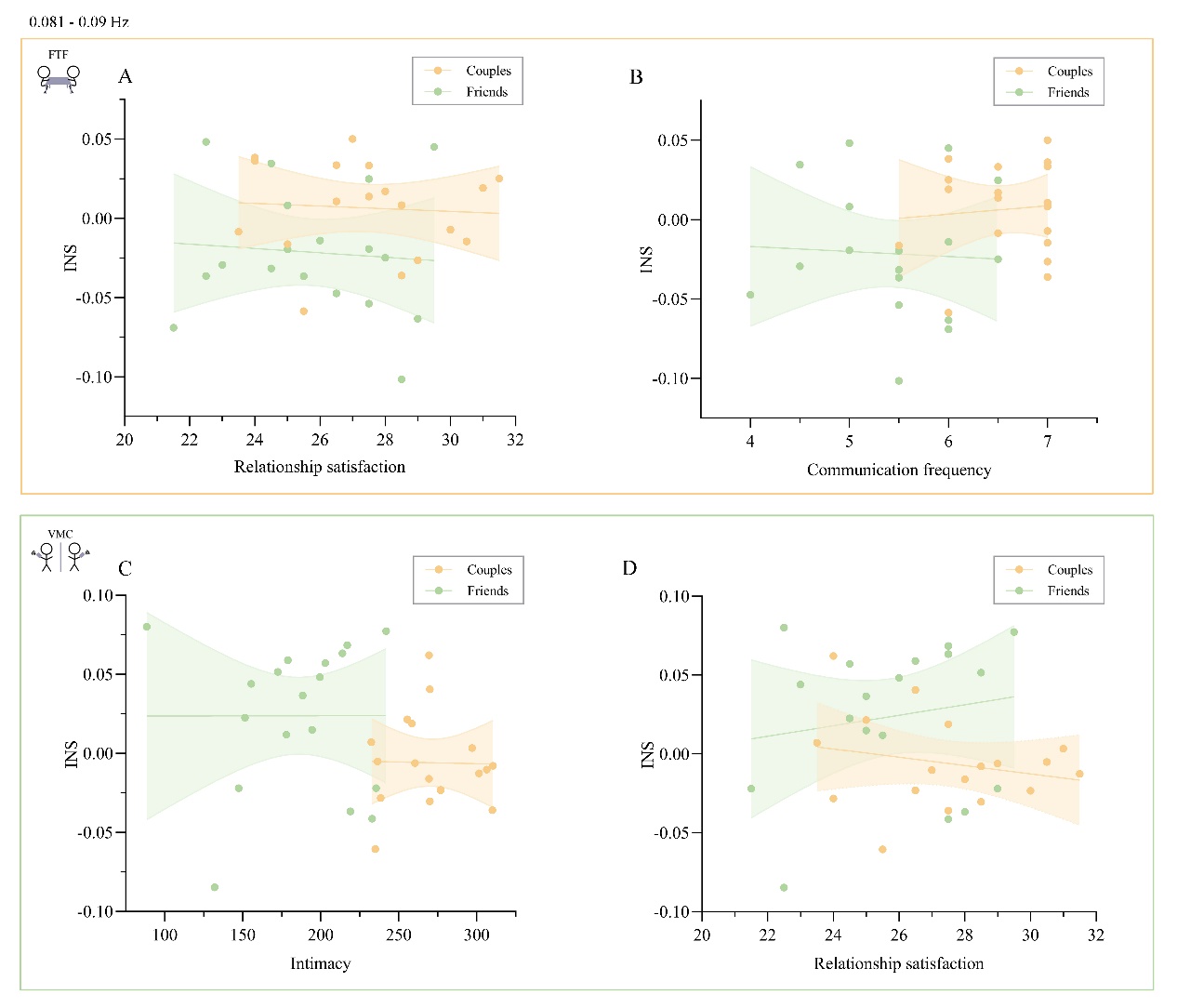
**Figure S3 High-frequency (0.081**–**0.090 Hz) dlPFC INS**

Correlations during FTF with (A) relationship satisfaction (*r* = 0.06, *p* = .729) and (B) communication frequency *(r* = 0.26, *p* = .124); during VMC with (C) intimacy (*r* = -0.29, *p* = .087), (D) relationship satisfaction (*r* = -0.11, *p* = .543).


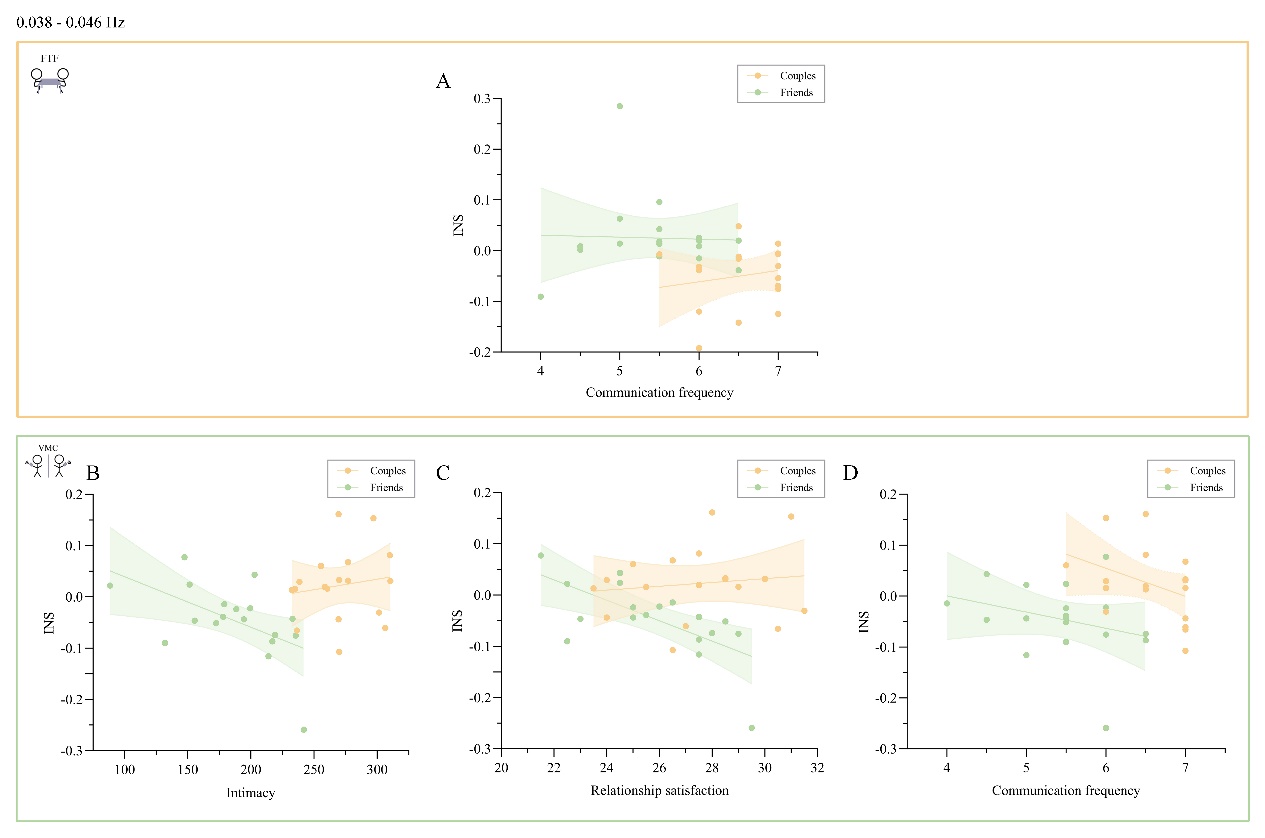
**Figure S4 Low-frequency (0.038–0.046 Hz) dlPFC INS**

Correlations during FTF with (A) communication frequency (*r* = -0.31, *p* = .067); during VMC with (B) intimacy (*r* = 0.20, *p* = .247), (C) relationship satisfaction (*r* = -0.08, *p* = .643), and (D) communication frequency (*r* = 0.10, *p* = .577).


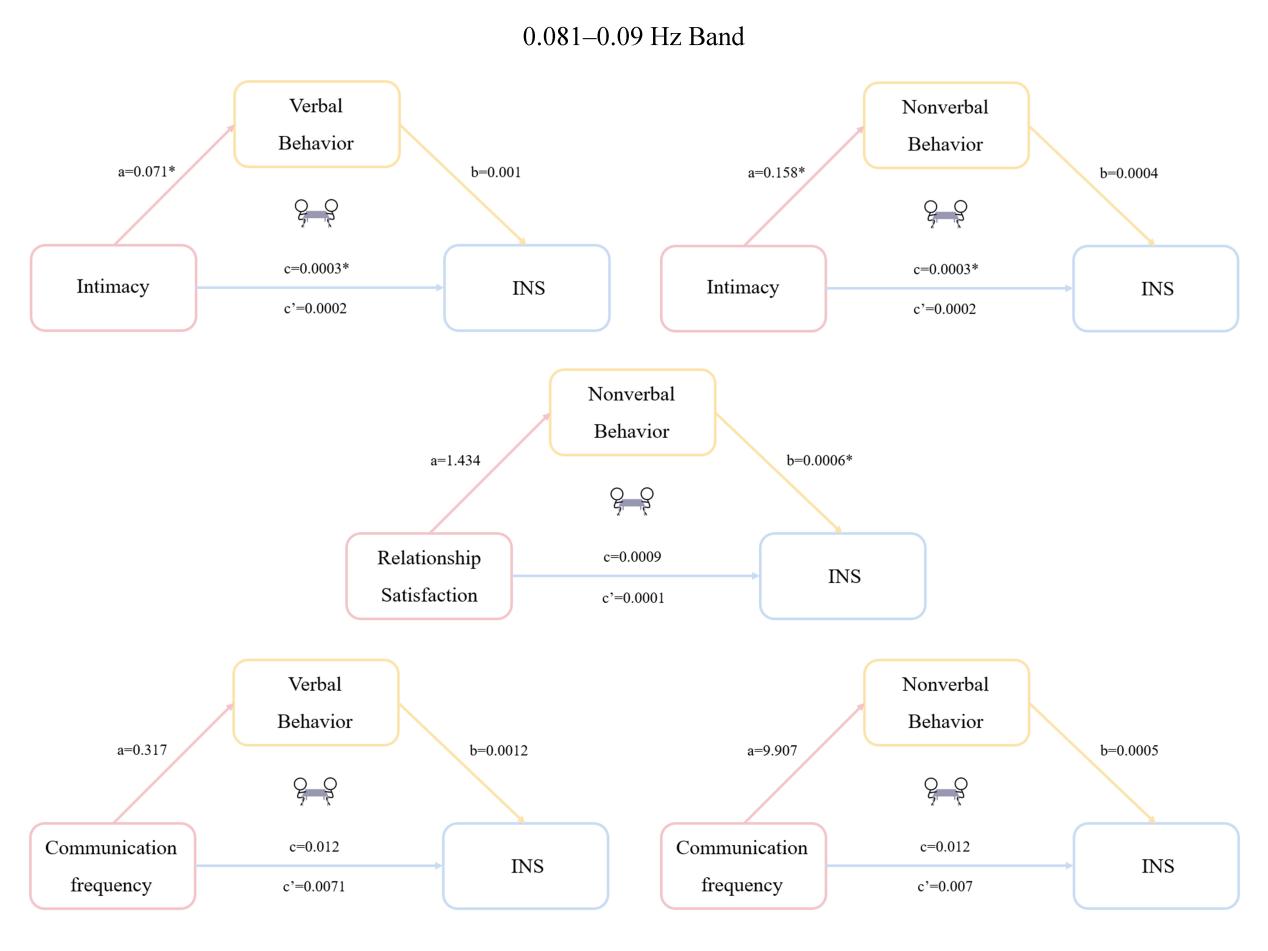


**Figure S5 Mediation model: FTF, high-frequency band**

Indirect effects of intimacy, relationship satisfaction, and communication frequency on high-frequency INS through verbal and non-verbal behaviour (no paths significant).


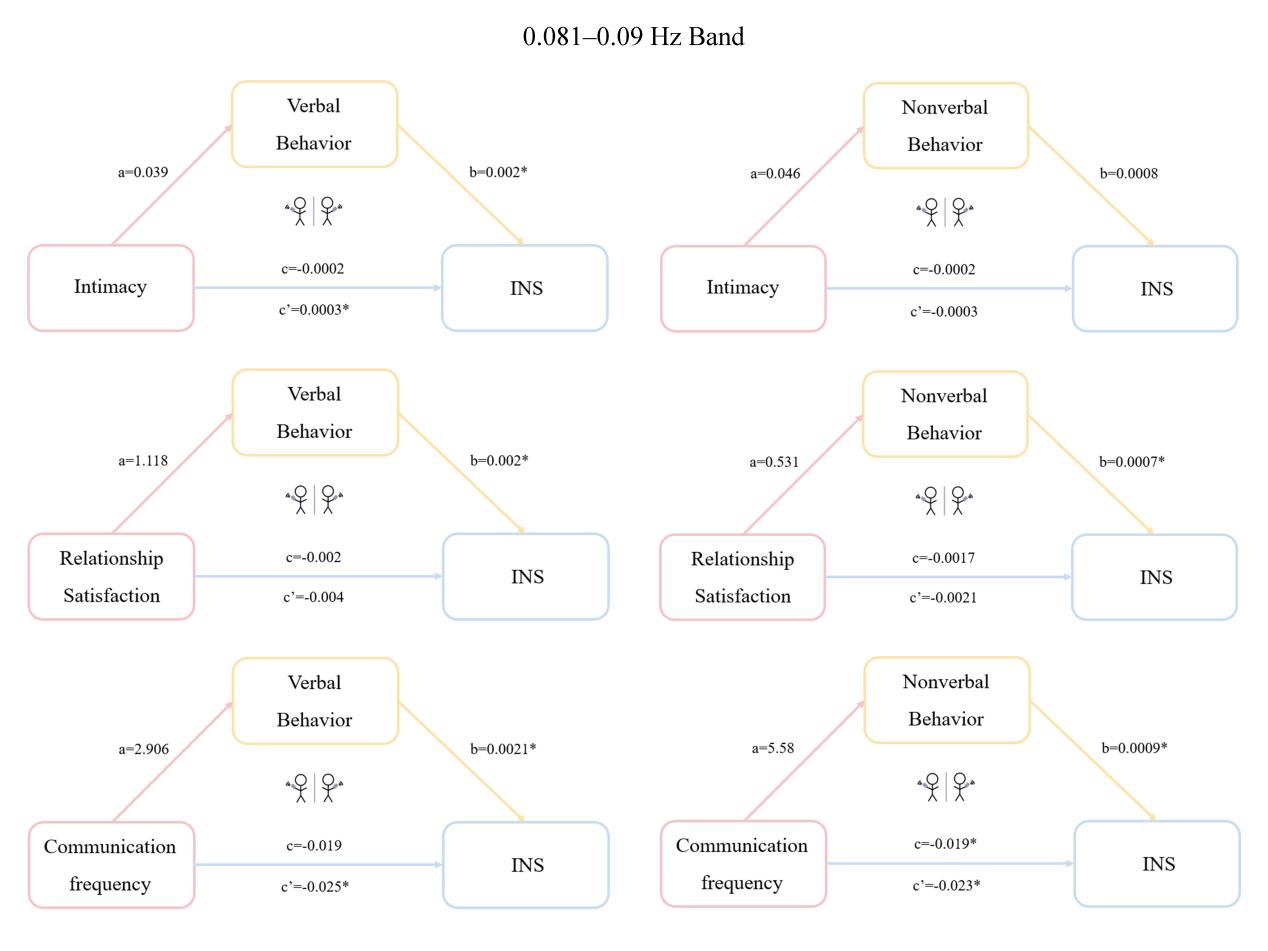


**Figure S6 Mediation model: VMC, high-frequency band**

Indirect effects of intimacy, relationship satisfaction, and communication frequency on high-frequency INS through verbal and non-verbal behaviour (no paths significant).


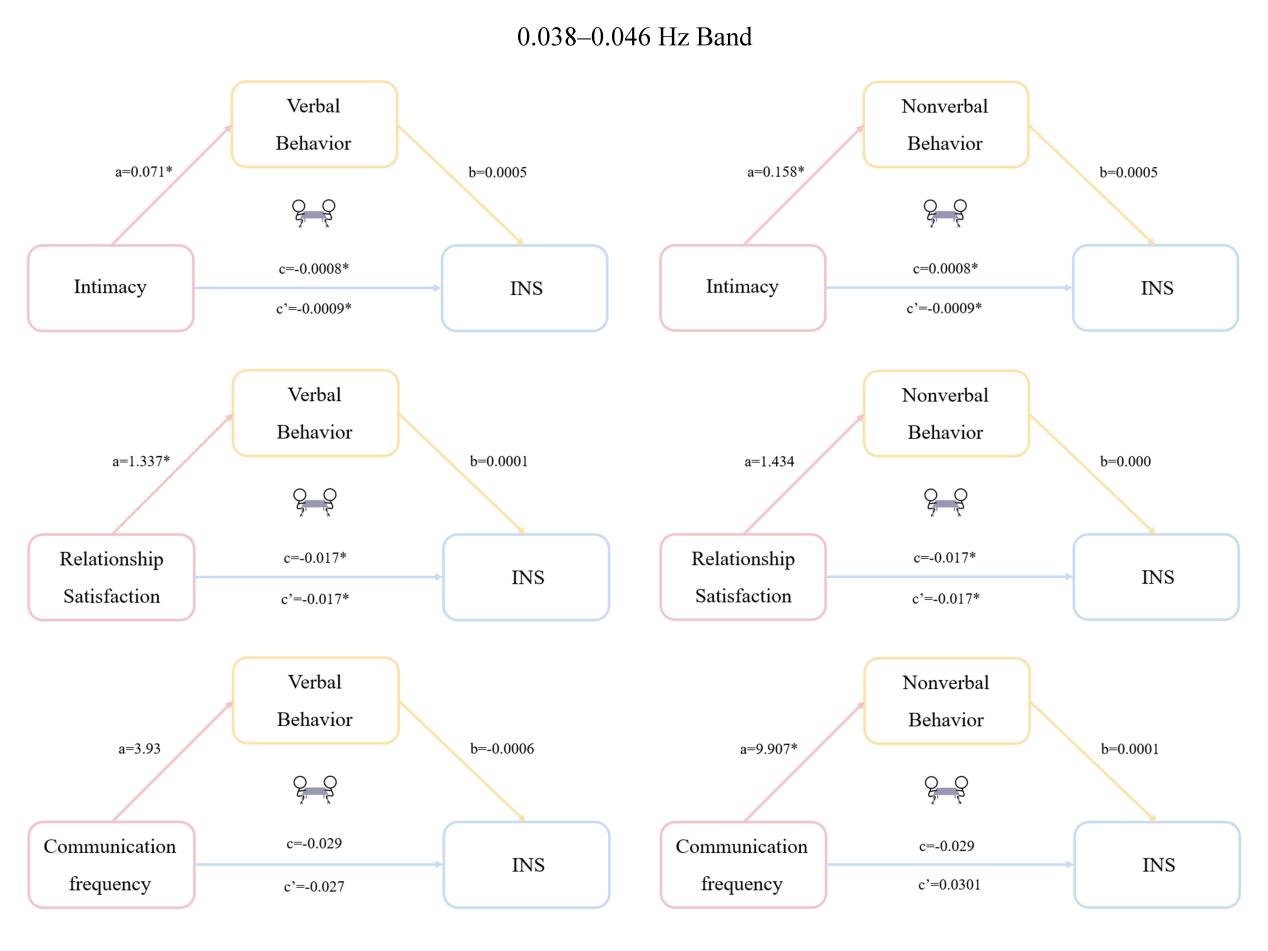


**Figure S7 Mediation model: FTF, low-frequency band**

Indirect effects of intimacy, relationship satisfaction, and communication frequency on high-frequency INS through verbal and non-verbal behaviour (no paths significant).


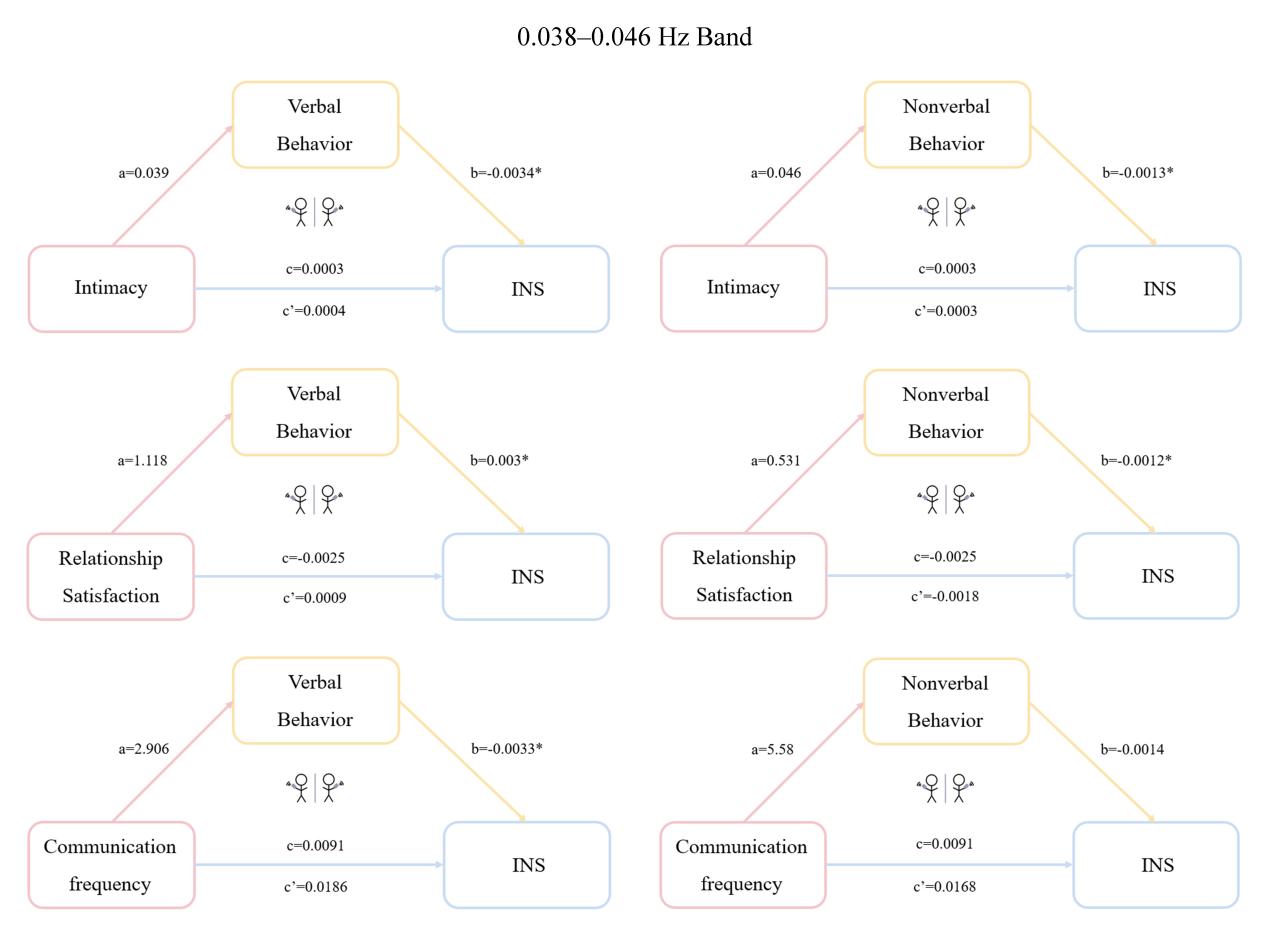


**Figure S8 Mediation model: VMC, low-frequency band**

Indirect effects of intimacy, relationship satisfaction, and communication frequency on high-frequency INS through verbal and non-verbal behaviour (no paths significant).
